# Distribution and Elimination of Deltamethrin Toxicity in Laying Hens

**DOI:** 10.3390/foods12244385

**Published:** 2023-12-06

**Authors:** Yiming Liu, Chunshuang Liu, Mingyue Han, Na Yu, Wen Pan, Jie Wang, Zhiying Fan, Wei Wang, Xiubo Li, Xu Gu

**Affiliations:** 1 National Feed Drug Reference Laboratories, Feed Research Institute, Chinese Academy of Agricultural Sciences, Beijing 100081, China; 2 Key Laboratory of Animal Antimicrobial Resistance Surveillance, Ministry of Agriculture and Rural Affairs, Feed Research Institute, Chinese Academy of Agricultural Sciences, Beijing 100081, China; 3 Laboratory of Quality & Safety Risk Assessment for Products on Feed-Origin Risk Factor, Ministry of Agriculture and Rural Affairs, Feed Research Institute, Chinese Academy of Agricultural Sciences, Beijing 100081, China; 4 Zhong Mu Institutes of China Animal Husbandry Industry Co., Ltd., Beijing 100095, China

**Keywords:** deltamethrin, GC-MS, laying hens, residue enrichment, residue elimination

## Abstract

Deltamethrin, an important pyrethroid insecticide, is frequently detected in human samples. This study aims to assess the potential effects of deltamethrin on human health and investigate the patterns of residue enrichment and elimination in 112 healthy laying hens. These hens were administered 20 mg·kg^−1^ deltamethrin based on their body weight. Gas chromatography-mass spectrometry (GC-MS) was used to investigate the residue enrichment pattern and elimination pattern of deltamethrin in the hens. The results indicated a significant increase in the concentration of deltamethrin in chicken manure during the treatment period. By the 14th day of administration, the concentration of deltamethrin in the stool reached 13,510.9 ± 172.24 μg·kg^−1^, with a fecal excretion rate of 67.56%. The pulmonary deltamethrin concentration was the second highest at 3844.98 ± 297.14 μg·kg^−1^. These findings suggest that chicken feces contain substantial amounts of deltamethrin after 14 days of continuous administration, and that it can easily transfer to the lungs. After 21 days of drug withdrawal, the residual concentration of deltamethrin in the fat of laying hens was 904.25 ± 295.32 μg·kg^−1^, with a half-life of 17 days and a slow elimination rate. In contrast, the lungs showed relatively low elimination half-lives of 0.2083 days, indicating faster elimination of deltamethrin in this tissue. These results highlight differences in the rate of deltamethrin elimination in different tissues during drug withdrawal. The fat of laying hens exhibited the highest residue of deltamethrin and the slowest elimination rate, while the lungs showed the fastest elimination rate. Moreover, deltamethrin was found to accumulate in the edible tissues of eggs and laying hens, suggesting that humans may be exposed to deltamethrin through food.

## 1. Introduction

Pyrethroids are synthetic modifications of natural pyrethroids that are used to kill insects by disrupting sodium channels in their nerves, ultimately affecting their nervous system function. Deltamethrin, being one of the pyrethroids, is frequently utilized for controlling agricultural insects and animal ectoparasites. Laying hens, being one of the most significant egg-producing animals, can be exposed to deltamethrin either by consuming grasses contaminated with deltamethrin or by having it applied on their skin to prevent ectoparasites. Deltamethrin, a fat-soluble molecule, can be absorbed through the skin and tends to accumulate in lipid-rich tissues of animals such as chicken, eggs, milk, and beef. These high-fat foods are commonly consumed due to their nutritional value, affordability, and accessibility, making it important to consider the potential exposure to deltamethrin through the consumption of such foods. The consumption of animal-based foods high in fat may lead to harm or various diseases in humans due to the bioaccumulation of these fats. Symptoms may include diarrhea, vomiting, and dermatitis [1,2].

The most commonly used methods for detecting pyrethroid pesticides in animal-derived food products are gas chromatography [3], gas chromatography-mass spectrometry (GC-MS) [4], high-performance liquid chromatography (HPLC) [5,6], and liquid chromatography-mass spectrometry (LC-MS) [7]. When analyzing deltamethrin in animal products, several factors can impede the process. These include the complex matrix of animal products, the compatibility of the instrument with the product being tested, and the trace amounts of deltamethrin present in the sample. Liquid chromatography (LC) has been reported to complicate the analysis of certain pyrethroids [8]. Therefore, gas chromatography (GC) [9,10] is frequently employed in conjunction with an electron capture detector, single-quadrupole mass analyzer (MS) [11], or triple-quadrupole mass analyzer in tandem MS/MS [12,13] to detect pyrethroid pesticides in animal-derived food products. On the basis of previous studies on the toxicology of deltamethrin in laying hens [14], we further studied the enrichment and elimination of the poison deltamethrin in the body of laying hens using gas chromatograph-mass spectrometry. The existing research on the toxicity and residue of deltamethrin in laying hens is currently limited. This study seeks to evaluate the potential impact of deltamethrin on human health and examine the patterns of residue enrichment and elimination of deltamethrin in laying hens. Gas chromatography-mass spectrometry was utilized to analyze deltamethrin levels in the blood, eggs, chicken manure, and tissues of the laying hens. These results are critical to improving the quality of commercial layer products, ensuring consumer safety, and assessing the risk of human exposure to deltamethrin.

## 2. Materials and Methods

### 2.1. Chemicals and Animals

Deltamethrin control (99.5%, batch number: 121720) and heptachlor epoxide control (99.4%, batch number: G121441) were purchased from Dr. Ehrenstorfer GmbH (Augsburg, Germany). The chemical raw material deltamethrin (98%) was obtained from Hubei Maoerwo Biological Medicine Co., Ltd. (Wuhan, China). Chromatographically pure acetonitrile, hexane, ethyl acetate, petroleum ether, and diethyl ether were purchased from Fisher Scientific (Fair Lawn, NJ, USA). Other chemical substances and reagents applied in this research were obtained from Shanghai Chemical Reagent Co., Ltd. (Shanghai, China). An FA25 tissue homogenizer was obtained from Shanghai Ferruck Company (Shanghai, China); a Fotector PlUS-60 solid phase extraction instrument was obtained from Reeko Instrument Co., Ltd. (Amoy, China).

Laying hens, weighing 1.0 ± 0.05 kg, were obtained from the Nan Kou pilot base of the Chinese Academy of Sciences of Agriculture in Beijing, China. The hens, which were 53 weeks old, were housed individually in cages and provided with unlimited access to drinking water and feed. Prior to the experiments, the hens were acclimated to the laboratory conditions for 1 week. The laboratory conditions maintained a temperature of 22 ± 2 °C and followed a 16/8 (light/dark) cycle. The animal experiments were conducted in accordance with the Animal Use and Care Committee of the Chinese Academy of Sciences of Agriculture (Beijing, China, number: FRI—CAAS—20180316).

### 2.2. Exposure Experiment and Sample Collection

Based on the daily feed intake of hens, the approximate daily intake of deltamethrin was 20 mg·kg^−1^ of body weight. Furthermore, the LD_50_ for deltamethrin in chickens exceeds 2000 mg·kg^−1^. The treated feed was prepared by dissolving deltamethrin in 1 mL of corn oil and then homogeneously mixing it. After 7 days of adaptation, a total of 112 laying hens were randomly assigned to 13 treatment groups (*n* = 8) and 1 control group (*n* = 8).

In the case of multiple-dose administration, the drug was administered once a day for 14 consecutive days at the same concentration as the single-dose administration. Laying hens were sacrificed on the 1st, 3rd, 7th, 10th, and 14th day during deltamethrin treatment, and their cockscomb, crop, heart, lung, liver, kidney, spleen, muscular stomach, glandular stomach, muscle, fat, blood, and manure were collected for testing. Laying hen eggs were collected for testing on the 7th, 10th, and 14th day during the deltamethrin treatment. During the withdrawal period, hens were sacrificed on multiple days including the 1st, 2nd, 3rd, 7th, 10th, 14th, 17th, and 21st day. Various organs and tissues such as cockscomb, crop, heart, lung, liver, kidney, spleen, muscular stomach, glandular stomach, muscle, fat, and blood were collected for testing purposes. Samples of eggs and manure were collected for testing on the 1st, 2nd, 3rd, 5th, 7th, 10th, 14th, and 17th day during the deltamethrin withdrawal period. Blood samples were taken from laying hens and centrifuged with 0.9% sodium chloride for 10 min at 3108 g to obtain plasma, which was stored at −40 °C until analysis.

### 2.3. Sample Preparation and Analysis

A 2.00 g ± 0.01 g tissue homogenate sample (obtained from layer tissue samples, hen manure, and eggs) was spiked with 100 μL of 1 μg·mL^−1^ epoxy heptachlor internal standard in a 50 mL centrifuge tube. The mixture was vortexed for 5 min and then combined with 15 mL of extraction solution. In this study, we utilized different concentrations of acidified acetonitrile for the extraction of deltamethrin in various samples. Specifically, a 0.6% acidified acetonitrile solution was employed for the extraction of deltamethrin in tissues, while a 0.4% acidified acetonitrile solution was used for the extraction of deltamethrin in eggs and blood. Moreover, a 6% acidified acetonitrile solution was utilized for the extraction of deltamethrin in manure. The resulting mixture was vortex mixed for 2 min. The extraction powder, which included anhydrous magnesium sulfate (2 g) and anhydrous sodium acetate (1.5 g), was added to the mixture. The solution was vortexed for 5 min, then centrifuged at 10,656× *g* for 10 min at 4 °C. The supernatant (5 mL) was collected and poured into a solid-phase extraction cleaner C18/PSA cartridge (500 mg/500 mg/6 mL) that had been pre-conditioned with 10 mL of acetonitrile. The percolated solution was collected into a 10 mL glass tube via gravity. The analyte was eluted from the cartridge using 5 mL acetonitrile and collected in the same tube. The solution was then concentrated to 1 mL under a gentle stream of nitrogen and filtered through a 0.22 μm nylon filter membrane before being analyzed with GC-MS.

Gas chromatography-mass spectrometry (GC-MS) analysis was conducted using an Agilent 7890A gas chromatograph, coupled with an Agilent 5975C quadrupole mass spectrometer. A HP-5 MS capillary column (5% phenyl 95% dimethylpolysiloxane, 30 m × 0.25 mm × 0.25 µm) was used to divide the chemical compounds. The injector temperature was set at 290 °C. For the splitless mode, the injection volume was 1.0 μL. The oven temperature program was set as follows: the oven temperature was held for 1 min at 70 °C, ramping at 20 °C· min^−1^ up to 250 °C, then ramping at 20 °C· min^−1^ up to 300 °C and dwelling for 6 min. The quadrupole temperature was set to 150 °C and the ion source temperature was set to 230 °C. Additionally, the MS inlet line and interface temperatures were set to 290 °C and 280 °C, respectively. The MS ionization energy was set at 70 eV (Table 1).

The analytical method was evaluated based on various parameters such as linearity, limit of detection (LOD), limit of quantification (LOQ), accuracy, precision, and matrix effects. The assessment was conducted in accordance with the criteria specified in European Commission SANTE/11312/2021 and Commission Decision 2002/657/EC [15,16]. In this study, deltamethrin standard and a 100 μL aliquot of 1 μg·mL^−1^ internal standard solution-epoxy heptachlor were added to different blank tissues, blank blood, blank eggs, and blank manure samples obtained from laying hens. These samples were then diluted to different concentrations ranging from 5 to 1000 μg·kg^−1^. After pretreatment, the samples were analyzed using GC-MS. The deltamethrin curve was created by plotting the peak area of deltamethrin against the peak area of epoxy heptachlor. The LOD and LOQ were determined as the minimum concentration of deltamethrin that produces a signal-to-noise ratio of ≥3 and ≥10, respectively.

For the accuracy and precision tests, samples of blank tissues, blank blood, blank eggs, and blank hen manure from laying hens were spiked with three concentrations of deltamethrin standard (25 μg·kg^−1^, 50 μg·kg^−1^, and 100 μg·kg^−1^), blank fat from laying hen was spiked with three concentrations of deltamethrin standard (250 μg·kg^−1^, 500 μg·kg^−1^, and 1000 μg·kg^−1^), and 100 μL aliquots of 1 μg·mL^−1^ of internal standard solution-epoxide heptachlor were analyzed in quintuplicate on three consecutive days. The purpose of this was to determine the closeness of agreement of the measurements compared to the analyzed concentrations (accuracy %) and repeated experiments (relative standard deviations (RSD)). The accuracy of the deltamethrin measurement was calculated by dividing the peak area of deltamethrin in the sample by the peak area of the internal standard in the sample. This value was then divided by the peak area of deltamethrin in the matrix standard solution, also divided by the peak area of the internal standard in the matrix standard solution. The final result was multiplied by 100%.

The accuracy and precision of GC-MS for analytes can be affected by matrix effects during assay analysis. Hence, it is crucial to assess these effects by analyzing samples from various tissues of blood and manure in laying hens. The matrix effects were determined by calculating the ratio of the slope of the standard curve made from matrix-matched mixed standard solutions to the slope of the standard curve made by mixing the standard solution with pure solvent and multiplying the result by 100%. To further investigate excretion characteristics through droppings, the excretion rate was determined. This rate was calculated by dividing the mass of deltamethrin found in the droppings by the mass of deltamethrin in the ingested feed.

### 2.4. Data Analysis

Means and standard errors (SEs) were calculated using Microsoft Excel (Microsoft Co., Redmond, WA, USA) for the analysis of elimination parameters in GraphPad Prism (ver.10) software (GraphPad Software Inc., San Diego, CA, USA). The study provided data on deltamethrin residue elimination parameters, such as the elimination rate and half-life.

## 3. Results and Discussion

### 3.1. Selection of Extraction Solvents

When extracting pyrethroid drugs from animal food, it is crucial to select an appropriate extraction solvent due to the complexity of the meat food matrix. Acetonitrile is a favorable choice as it has high solubility, less matrix interference, strong versatility, and good extraction effect. Moreover, previous acetonitrile studies have also shown the same results [17,18,19]. In this study, acetonitrile was selected as the extractant due to its low boiling point. However, it should be noted that acetonitrile has been reported to increase the pressure within the GC-MS system and may not be compatible with the instrument. Additionally, the use of a solvent with a high boiling point may result in the loss of highly volatile analytes. Therefore, it is important to choose a solvent with a sufficiently low boiling point [20]. This study aimed to test and optimize the extraction solvent acidification using various concentrations of acetic acid (0.1%, 0.2%, 0.4%, 0.6%, 1%, 2%, 4%, and 6%) to ensure consistent and reliable recoveries of deltamethrin. The optimal proportion of acidified acetonitrile for extracting deltamethrin from tissue samples was found to be 0.6%, resulting in a recovery rate of 96.10 ± 10.56%. For extracting deltamethrin from eggs and blood samples, the best method was using 0.4% acidified acetonitrile, with recoveries of 96.83 ± 5.38% and 90.92 ± 2.95%, respectively. The use of 6% acidified acetonitrile proved to be effective in extracting deltamethrin from stool samples, with a recovery rate of 90.92 ± 2.95% (Table 2).

### 3.2. Method Validation

In the concentration range of 5–1000 μg·kg^−1^, deltamethrin’s peak area exhibited an excellent linear relationship with the concentration (Table 3).

The sensitivity of the method was assessed by determining the limits of detection (LOD) and quantitation (LOQ), which were found to be 5 μg·kg^−1^ and 15 μg·kg^−1^, respectively (Table 3). These values were lower than those reported in previous studies [21,22], indicating that the method had a good detection performance.

We obtained accuracy and precision results for the detection of deltamethrin at three different spiking concentrations: 25, 50, and 100 μg·kg^−1^ (or 250, 500, and 1000 μg·kg^−1^). The accuracy of this method was evaluated based on the average recovery rate of determination at three different concentrations. The intra-day recovery rate and inter-day recovery rate of determination were found to be between 81.57–112.34% and 82.19–112.34% at a concentration of 25 μg·kg^−1^, respectively. Similarly, at a concentration of 50 μg·kg^−1^, the intra-day recovery rate and inter-day recovery rate of determination were found to be between 86.16–107.32% and 84.68–110.39%, respectively. The study found that the intra-day and inter-day recovery rates of determination were within the ranges of 85.42–106.07% and 87.73%–105.60%, respectively, at a concentration of 100 μg·kg^−1^. At a concentration of 250 μg·kg^−1^, the intra-day and inter-day recovery rates of determination were 118.32% and 117.19%, respectively. The study found that the intra-day recovery rate and inter-day recovery rate of determination were 106.51% and 106.80% at 500 μg·kg^−1^, respectively. Similarly, the intra-day recovery rate and inter-day recovery rate of determination were 112.54% and 111.14% at 1000 μg·kg^−1^. The recoveries mostly fell within the recommended range of 70–110% as suggested by the Codex Alimentarius [23]. The high recoveries (111.14% and 112.54%) observed may be attributed to the presence of endogenous interfering compounds [4], To assess precision, repeatability (inter-day) and reproducibility (intra-day, over three successive days) were measured at three spiking levels. Intra-day and inter-day RSDs were calculated and found to range of 2.78–16.88% and 2.73–19.70%, respectively, over the three successive days at the three spiking levels (Table 4). Two instances in the intra-day and four instances in the inter-day surpassed an RSD of 15%. The study found that in most cases, the RSD (Relative Standard Deviation) was less than 15%. The results indicate that the method used in the study had better repeatability, reproducibility, and recovery than similar studies conducted under equal spiked concentrations [24,25].

Matrix effects are a common occurrence in GC-MS analysis of complex samples, often resulting in significant matrix enhancement effects [26,27,28]. The impurities present in the sample matrix compete with the target compound molecules for the active site at the inlet or column head during the instrumental detection process. This competition reduces the chance of interaction between the target and the active site, resulting in a higher response value for the same content of the target compound in the actual sample than in the pure solvent specimen. Matrix effects in GC-MS methods can be influenced by various factors such as the chemical structure, concentration, and nature of the target being measured, as well as the type, concentration, acidity, and state of the matrix. To reduce these effects, several techniques have been reported including matrix-matching correction methods [28], isotope internal standard methods [29,30], addition of protectants/masking methods [31], and improvements to the injection technique. Matrix matching correction was employed in this study to mitigate the impact of matrix effects on the analysis results. The findings revealed that, except for blood samples which showed matrix inhibition effects, all other samples exhibited matrix enhancement effects. Among these, deltamethrin demonstrated the least enhancement effect in fat (Table 5). Our study effectively eliminated or compensated for the effects of matrix effects by employing matrix-matched standard solutions.

### 3.3. Study on the Enrichment Pattern of Deltamethrin in Laying Hens

The swift absorption of deltamethrin following oral administration and its subsequent distribution to different organs and tissues via the bloodstream have been reported [32]. The study focuses specifically on the enrichment and elimination of deltamethrin in laying hens, providing valuable information for future related research. In this study, we examined the distribution and residual changes of deltamethrin in 112 healthy laying hens. The hens were given a specific dose of the drug based on their body weight, and their samples were processed using pretreatment methods and analyzed using GC-MS.

The distribution of the drug in tissues and eggs is influenced by various factors, including tissue blood supply, drug permeability in tissues, and the presence of binding sites. The physical and chemical properties of the drug, such as its lipophilicity, also play a significant role [33,34]. During the deltamethrin treatment period, it was observed that the concentration of deltamethrin in the crop reached its highest level on the 10th day. However, by the seventh day, the concentration of deltamethrin in the crop decreased rapidly. Among the analyzed samples, the liver exhibited the lowest concentration of deltamethrin. Interestingly, the concentration of the drug in the liver gradually increased and reached its peak on the 10th day. This increase in concentration could be attributed to the liver’s tendency to accumulate fat deposits [35]. Subsequently, the concentration gradually decreased. This accumulation of deltamethrin in the liver may be attributed to its stronger affinity for adipose tissue [36]. During the deltamethrin treatment period, the concentration of deltamethrin in the lungs peaked on the first day. The higher concentration of deltamethrin in the lung after oral administration in this study may be attributed to the capture of deltamethrin in the pulmonary microcirculation [37]. The concentration of deltamethrin in the kidney initially increases slowly and then decreases rapidly, which may be attributed to the kidney’s role as an excretory organ for deltamethrin [38]. A significant increase in cardiac activity was observed between the first day and seventh day, with the highest concentration of deltamethrin in the heart on the fourteenth day. The study findings revealed that deltamethrin exhibited a propensity to accumulate in the heart tissues of laying hens. The study observed a distinct ‘double peak’ trend in deltamethrin concentrations in the glandular stomach. The concentrations reached their highest level on the third and tenth days, after which they gradually declined. There was a significant decrease in the concentration of muscular stomach enrichment from the first day to the seventh day, reaching the lowest level on the seventh day. These findings suggest that deltamethrin is effectively absorbed from the gastrointestinal tract. The observed higher concentration of pyrethroids in corn oil carriers in the high-dose group could be a contributing factor to this absorption (Table 6) [39].

During the deltamethrin treatment period, the concentration of deltamethrin in fat was measured to be 3565.64 ± 74.78 μg·kg^−1^ on the 3rd day and 3618.77 ± 253.32 μg·kg^−1^ on the 10th day during the deltamethrin treatment period (Table 6). This study found that adipose tissue played a major role in the systemic predisposition to deltamethrin on the 21st day during the withdrawal period. The concentration of deltamethrin in adipose tissue was considerably higher compared to blood and other tissues, indicating that deltamethrin tends to accumulate predominantly in fat. During the 17th day of the withdrawal period, the adipose tissue samples had the highest residual level of deltamethrin (Table 7), which aligns with previous studies that have shown deltamethrin to be extensively distributed in fat [40,41,42]. The concentration of deltamethrin in blood gradually increased from the first day to the seventh day during the deltamethrin treatment period, but then showed significant fluctuations. The lowest concentration of deltamethrin in blood was observed on the 10th day. This trend may be related to the rapid absorption of deltamethrin through the lipid membranes of red blood cells after oral administration and its arrival to different tissues/organs through the diffusion cycle [32]. The change in deltamethrin concentration in the blood of laying hens was the same as that in the blood of pigs, which showed a trend of rapid rise and then slow decline [43]. During the deltamethrin treatment period, the concentration of deltamethrin in hen manure significantly increased, indicating that most of the deltamethrin was excreted through manure, with an excretion rate of 67.56%. Excretion plays a crucial role in the elimination of deltamethrin in laying hens. The findings of this study are consistent with previous research, suggesting that deltamethrin was not easily absorbed from the gastrointestinal tract of laying hens [44,45,46].

During the deltamethrin treatment period, the highest concentration was found in chicken manure on the 14th day, while the lowest concentration was found in the muscle stomach on the same day. Similarly, during the withdrawal period, the highest deltamethrin residue was detected in fat on the 21st day, whereas the lowest residue was found in the liver on the same day. These findings are consistent with previous studies that have also observed relatively high concentrations of deltamethrin residues in eggs after laying hens stop consuming oral deltamethrin [47,48]. Studies have found that deltamethrin tends to accumulate in the lungs, fat, and crop tissue. However, it is rapidly eliminated in the liver, which may be attributed to the liver’s role as a major organ for metabolizing and detoxifying pesticides. This finding is consistent with previous studies [49,50]. Previous studies have demonstrated that deltamethrin can accumulate in various tissues of chickens, including the heart, kidney, lung, liver, blood, fat, and eggs, leading to toxicological effects. Our study further supports this finding, indicating that deltamethrin tends to accumulate more in tissues with higher fat content [51,52,53]. Studies have shown that birds possess a highly efficient pyrethroid metabolism mechanism [54], However, pyrethroid residues have been found in various tissues and organs of birds, indicating that these chemicals can enter the food chain and potentially pose health risks to both birds and humans [55].

### 3.4. Study on the Elimination Pattern of Deltamethrin in Laying Hens

This study investigated the elimination of deltamethrin residues in various tissues, including eggs, chicken feces, and laying hens’ blood, during the withdrawal period. The degradation rate of deltamethrin was found to be faster in the muscles and muscle stomach, with no detectable traces of the substance on the first day. On the second day, the concentration of deltamethrin in the glandular stomach was below the detection limit of 5 μg·kg^−1^. However, in the comb, crops, and spleen, the degradation process was slower, leading to the presence of residues even after the third day. After the 21st day, the metabolism of deltamethrin in various organs, such as the liver, slowed down, resulting in the accumulation of significant residue amounts.

As per the regulations set by the Codex Alimentarius Commission [56], the permissible amount of deltamethrin residue in poultry muscle and eggs is limited to 30 μg·kg^−1^. However, in the case of poultry liver and kidney, the maximum residue limit is set at 50 μg·kg^−1^. The maximum residue limit for poultry fat is comparatively higher, at 500 μg·kg^−1^. This study found that deltamethrin residues in fat and eggs exceeded the maximum residue limits set by the Codex Alimentarius Commission (Table 7). During the withdrawal period, residue levels in blood, chicken manure, lungs, heart, kidneys, and liver showed a significant reduction on the first day. The concentration of deltamethrin residue in each tissue decreased over time. However, on the 21st day, substantial amounts of deltamethrin were still detected in the lungs, kidneys, and adipose tissue. Moreover, all of these tissue samples surpassed the residue levels set by the Codex Alimentarius Commission (Table 7). Hence, it is crucial to monitor the levels of deltamethrin residues in lungs, eggs, muscles, liver, kidneys, and adipose tissue simultaneously, as these are the primary areas where such residues are commonly found. The results indicated that on the 14th day, during the deltamethrin treatment period, the liver exhibited a lower deltamethrin residue compared to other tissues, with a concentration of 126.95 ± 19.27 μg·kg^−1^. This observation suggests that the liver plays a crucial role in the metabolic transformation of deltamethrin [57,58]. During the deltamethrin treatment period, the concentration of deltamethrin in blood on the 14th day was lower compared to that in lung, fat, and chicken’s crown, but higher than in other tissues (385.61 ± 78.36 μg·kg^−1^). This variation may be attributed to the rapid absorption of deltamethrin, which binds to the lipid membranes of red blood cells, enabling its diffusion into various tissues and organs [32].

After the last dose, the half-life of deltamethrin was observed to be lower in hen manure, eggs, blood, and other tissues compared to fat. The study also found that the half-life of deltamethrin in hen manure was 0.4451 days. This suggests that the elimination process of deltamethrin may be influenced by the degree of gastrointestinal absorption [59]. The elimination half-life of deltamethrin in droppings was found to be only 0.4451 days. This short half-life could be attributed to incomplete absorption from the feed during exposure or the uptake of the pesticide into hen tissues with higher lipid contents [60], such as fat [61]. There was a gradual decrease in the concentration of deltamethrin in fat, indicating its ability to accumulate in fat. Deltamethrin residues in the fat of laying hens, being lipophilic, had a relatively long elimination half-life of 17 days, consistent with previous research [62]. Deltamethrin, a type of pyrethroid, exhibits lipophilic properties and tends to accumulate in lipid-rich tissues. Similar findings have been observed in studies involving mice [63] and bovines [64]. This phenomenon may be attributed to the fact that fat primarily consists of triglycerides, which have a tendency to accumulate lipophilic substances. This study suggests that the long half-life of deltamethrin in fat, along with its rapid metabolism in the lungs and liver, may inhibit the redistribution of fatty tissue to other tissues as a secondary source. In our experiments, we observed that the elimination half-life of deltamethrin in the blood was 8.217 days. It was found that the metabolism of deltamethrin in the blood was slower compared to other tissues. This could be attributed to a higher redistribution of deltamethrin from other tissues into the blood, which is consistent with the findings for other pyrethroids [65]. Due to its lipophilic nature, deltamethrin has an elimination half-life of 9.294 days in eggs. This phenomenon could be attributed to the higher presence of phospholipids and cholesterol in eggs. The liver and lung showed relatively fast elimination rates, with half-lives of 2.258 days and 3.328 days, respectively. The elimination rate of deltamethrin in eggs was relatively slow, which aligns with previous findings (Table 8) [62,66].

In conclusion, our research found that the concentration of deltamethrin in chicken feces significantly increased during the administration of laying hens. On the 14th day of administration, the concentration of deltamethrin in feces was 13,510.9 ± 172.24 μg·kg^−1^, with a fecal excretion rate of 67.56%. The lung deltamethrin concentration was 3844.98 ± 297.14 μg·kg^−1^. These findings indicate that chicken feces contained high levels of deltamethrin after 14 days of continuous administration, and it easily transferred to the lungs. After 21 days of drug withdrawal, the residual concentration of deltamethrin in the fat of laying hens was 904.25 ± 295.32 μg·kg^−1^, with a half-life of 17 days and a slow elimination rate. On the other hand, the lung had a relatively low elimination half-life of 0.2083 days, suggesting that deltamethrin is eliminated more rapidly in this tissue. Furthermore, deltamethrin was found to accumulate in the edible tissues of eggs and laying hens, indicating potential exposure to deltamethrin through food for humans. Given the widespread use of pyrethroids worldwide, there is significant concern regarding the potential risk of human exposure to food residues. It is crucial to conduct a cumulative risk assessment of pesticides in animals to protect consumer health and ensure the profitability of food producers. Our study discovered that long-term exposure to deltamethrin led to a gradual accumulation of deltamethrin in vital organs of laying hens, with high residual levels still present in tissues even 21 days after exposure. The presence of deltamethrin residue has a highly detrimental impact on animal food safety. Therefore, it is imperative to control the misuse of deltamethrin in laying hen production and strictly adhere to proper application practices. This study enhances our understanding of the toxicity of deltamethrin in laying hens and provides valuable insights into the safe utilization of deltamethrin in poultry production. By avoiding dietary exposure to deltamethrin residue levels in edible tissues of laying hens, both animal food safety and human health can be ensured. Currently, there is limited information available on the risk assessment of exposure to deltamethrin residues in laying chicken tissue, making the original findings of this study a significant contribution to public health. The methodology established in this study can also be applied to analyze household pesticide residues, providing a reference for future research in this field.

The use of pesticides worldwide has significantly increased in recent decades, primarily due to changes in agricultural practices and the adoption of intensive farming [67,68]. Birds can also be exposed to pesticides through pest management methods like mite and tick control, as well as through the food chain [69]. Egg-layer food is one of the most widely consumed food items globally. However, there is growing concern about its contamination with pharmacologically active substances used for disease control or to enhance poultry growth [70]. One such contaminant is deltamethrin, which is commonly used as a household insecticide and is highly effective in controlling mosquito-borne diseases. Deltamethrin is extensively used in agriculture and can be transferred to animals through their consumption of contaminated plants. Heavy use of deltamethrin can have serious ecological implications and potential health risks, including acute, subacute, and chronic poisoning in both humans and animals [70]. The general population is primarily exposed to pesticides through the consumption of contaminated food and water, making them the main source of deltamethrin exposure due to its rapid absorption when ingested [71]. Human exposure to deltamethrin, whether through skin contact or ingestion, can lead to acute intoxication, characterized by symptoms such as rash, blistering, sore throat, nausea, abdominal pain, and even loss of consciousness [72]. Deltamethrin residues have been detected in the edible tissue of laying hens, making them a significant source of human exposure to deltamethrin contamination. As deltamethrin is primarily metabolized by liver microsomal enzymes and excreted by the kidneys, its metabolites tend to accumulate in these organs, causing the most significant chemical impact. It is important to note that there may be notable variations in metabolic patterns between poultry and mammals. Hence, it is crucial to obtain species-specific metabolic and residue data to accurately evaluate the potential risk to consumer safety associated with residues in poultry-derived food products. Additionally, this data will aid in promoting the appropriate utilization of deltamethrin in laying hens. Gathering data on the enrichment and elimination of these toxins in the edible tissue of laying hens will make a valuable contribution to the livestock industry, aiding in the determination of residual risks and the implementation of measures to prevent animal poisoning. This can be achieved by studying various parameters related to deltamethrin poison elimination.

## Figures and Tables

**Table 1:** The mass spectrometric parameters of heptachlor methane and deltamethrin.

Compound Name	Quantitative Ion	Qualitative Ion 1	Qualitative Ion 2	Collision Energy (eV)
Epoxy heptachlor	353	355	351	70
Deltamethrin	181	172	174	70

**Table 2:** Extraction recovery of deltamethrin from samples with different ratio acidification (*n* = 3).

Acidification Ratio	Tissue	Eggs	Blood	Hen Manure
0.1%	135.75 ± 6.72%	187.01 ± 6.23%	117.13 ± 9.19%	300.59 ± 10.61%
0.2%	137.20 ± 9.85%	150.07 ± 7.78%	98.42 ± 5.24%	
0.4%	149.18 ± 2.32%	96.83 ± 5.38%	90.92 ± 2.95%	
0.6%	96.10 ± 10.56%	76.56 ± 8.47%	79.50 ± 4.22%	
1%	62.45 ± 7.09%	49.18 ± 9.94%	68.64 ± 6.94%	183.42 ± 5.42%
2%				332.43 ± 7.36%
4%				117.79 ± 10.78%
6%				100.10 ± 7.78%

**Table 3:** Summary of method validation results of deltamethrin in different substrates.

Sample	Linear Equation	Correlation Coefficient	The Limits of Detection	The Limits of Quantitation
Cockscomb	y = 0.8208x + 0.3344	0.9996	5 μg·kg^−1^	15 μg·kg^−1^
Crop	y = 0.8508x + 0.2958	0.9946		
Heart	y = 0.8215x + 0.3817	0.9996		
Lung	y = 0.8111x + 0.1568	0.9993		
Liver	y = 0.8291x + 0.0936	0.9978		
Kidney	y = 0.8447x + 0.2332	0.9986		
Spleen	y = 0.1951x + 0.0377	0.9926		
Muscular stomach	y = 0.7359x + 0.1923	0.9954		
Glandular stomach	y = 0.8583x + 0.1736	0.9919		
Muscle	y = 0.6667x + 0.1524	0.9961		
Fat	y = 0.6084x + 0.1326	0.9987		
Eggs	y = 0.4168x + 0.1824	0.9938		
Blood	y = 0.3592x + 0.0932	0.9992		
Hen manure	y = 0.22x + 0.2888	0.9912		

**Table 4:** Average recovery and RSD values of deltamethrin in different samples (*n* = 5).

**Concentration** **(μg·kg^−1^)/Sample**	**Intra-Day Recovery Rate (%)**			**Intra-Day RSD** **(%)**			**Inter-Day Recovery Rate (%)**			**Inter-Day RSD** **(%)**		
	**25**	**50**	**100**	**25**	**50**	**100**	**25**	**50**	**100**	**25**	**50**	**100**
Cockscomb	96.27	99.90	103.57	10.35	14.45	13.14	96.58	98.34	102.39	8.77	15.64	15.03
Crop	86.68	88.69	89.68	9.70	7.67	10.05	93.08	91.58	91.57	8.54	9.61	10.90
Heart	81.57	86.16	91.70	11.88	8.40	8.86	82.19	84.68	90.38	10.92	8.89	9.57
Lung	98.64	107.01	106.07	9.01	12.22	7.50	94.00	97.13	100.10	7.83	19.70	7.78
Liver	96.34	92.68	99.31	7.38	6.82	7.75	94.89	100.79	98.37	11.97	10.24	13.63
Kidney	83.07	91.44	92.51	9.11	13.24	13.17	84.17	96.82	94.37	9.63	18.20	11.29
Spleen	89.35	106.65	96.98	7.62	7.92	9.61	82.66	104.41	100.20	6.46	6.35	10.60
Muscular stomach	91.10	95.28	95.33	15.82	4.61	12.47	96.22	98.96	97.22	14.59	7.30	12.30
Glandular stomach	92.81	94.03	98.12	16.88	5.10	5.17	94.01	90.82	98.19	12.75	6.81	6.64
Muscle	95.44	88.40	101.56	2.78	10.55	11.55	99.16	90.44	99.96	2.73	9.82	10.34
Eggs	97.67	100.26	85.42	14.39	14.78	7.06	97.03	102.64	87.73	11.10	16.00	6.85
Blood	89.02	107.32	103.59	13.51	9.96	10.43	89.02	110.39	105.60	17.75	9.36	13.95
Hen manure	112.34	91.90	100.09	11.44	12.16	10.02	112.34	93.35	100.09	11.44	14.38	10.03
**Concentration** **(μg·kg^−1^)/Sample**	**Intra-Day Recovery Rate (%)**			**Intra-Day RSD** **(%)**			**Inter-Day Recovery Rate (%)**			**Inter-Day RSD** **(%)**		
	**250**	**500**	**1000**	**250**	**500**	**1000**	**250**	**500**	**1000**	**250**	**500**	**1000**
Fat	118.32	106.51	112.54	11.15	7.12	5.10	117.19	106.80	111.14	11.32	6.24	7.06

**Table 5:** The matrix effects of deltamethrin in different substrates.

Sample	Matrix Effects (%)
Cockscomb	118.99
Crop	127.01
Heart	119.18
Lung	116.64
Liver	121.21
Kidney	125.37
Spleen	112.4
Muscular stomach	96.34
Glandular stomach	129.00
Muscle	77.88
Fat	62.32
Eggs	11.20
Blood	−4.16
Hen manure	41.30

**Table 6:** Concentration monitoring of deltamethrin in laying hens.

Sample	Drugs Administration Period (μg·kg^−1^)				
	1 d	3 d	7 d	10 d	14 d
Eggs	23.35 ± 2.87	112.34 ± 24.28	603.55 ± 37.82	491.29 ± 61.22	443.16 ± 40.40
Crop	5406.16 ± 717.36	2377.14 ± 341.38	3441.86 ± 56.22	4077.98 ± 640.39	2377.14 ± 341.38
Cockscomb	560.98 ± 38.41	277.10 ± 23.98	347.18 ± 42.71	217.19 ± 4.13	121.66 ± 17.9
Spleen	ND	179.88 ± 34.18	267.65 ± 15.94	276.22 ± 52.10	284.47 ± 8.77
Liver	91.96 ± 22.52	110.24 ± 37.72	124.73 ± 9.13	146.99 ± 53.82	126.95 ± 19.22
Lung	8553.83 ± 1133.94	3749.72 ± 137.42	3474.30 ± 526.56	5670.60 ± 1382.27	3844.98 ± 297.14
Kidney	412.18 ± 22.81	418.11 ± 30.69	453.62 ± 40.36	434.98 ± 46.31	238.20 ± 31.65
Heart	109.68 ± 14.95	131.82 ± 9.32	204.09 ± 56.85	244.44 ± 39.54	261.39 ± 78.26
Glandular stomach	611.43 ± 39.33	770.91 ± 172.00	288.39 ± 36.00	488.88 ± 97.87	273.19 ± 77.97
Muscular stomach	220.68 ± 43.13	152.48 ± 20.86	90.31 ± 5.77	151.96 ± 16.03	111.28 ± 14.50
Muscle	264.77 ± 46.53	659.92 ± 19.70	333.87 ± 66.99	467.15 ± 68.49	192.58 ± 65.91
Fat	830.70 ± 174.22	3565.64 ± 74.78	3444.05 ± 61.86	3618.77 ± 253.32	2666.10 ± 5.13
Blood	423.19 ± 66.42	560.46 ± 16.29	622.45 ± 34.15	241.70 ± 46.92	385.61 ± 78.36
Hen manure	5191.89 ± 507.97	6749.18 ± 480.80	7089.48 ± 1602.73	11,036.51 ± 1988.69	13,510.9 ± 172.24

Notes: “ND” indicates that less than LOQ.

**Table 7:** Elimination of deltamethrin residues in laying hens (μg·kg^−1^).

**Drugs** **Withdrawal** **Period**	**Muscle**	**Muscular Stomach**	**Glandular Stomach**	**Crop**	**Spleen**	**Cockscomb**	**Liver**
0 d	192.58 ± 65.91	111.28 ± 14.50	273.19 ± 77.97	2377.14 ± 341.38	284.47 ± 8.77	126.85 ± 17.93	126.95 ± 19.27
1 d	ND	ND	36.69 ± 4.61	148.08 ± 33.83	81.75 ± 4.46	102.24 ± 58.95	10.50 ± 1.57
2 d	-	-	23.39 ± 0.14	132.17 ± 22.47	96.25 ± 32.48	97.96 ± 0.00	12.74 ± 1.88
3 d	-	-	-	126.59 ± 33.15	58.30 ± 4.08	75.29 ± 34.89	11.75 ± 0.57
7 d	-	-	-	-	-	-	7.99 ± 1.73
10 d	-	-	-	-	-	-	10.71 ± 1.09
14 d	-	-	-	-	-	-	10.59 ± 0.98
17 d	-	-	-	-	-	-	9.76 ± 1.47
21 d	-	-	-	-	-	-	9.55 ± 0.92
**Drugs** **Withdrawal** **Period**	**Heart**	**Kidney**	**Lung**	**Fat**	**Blood**	**Hen manure**	**Eggs**
0 d	261.39 ± 78.26	238.20 ± 31.65	3844.98 ± 297.14	2666.10 ± 5.13	385.61 ± 78.36	13,510.90 ± 172.24	443.16 ± 40.40
1 d	90.15 ± 7.02	72.02 ± 16.59	129.38 ± 6.88	4017.73 ± 256.34	216.73 ± 86.30	2552.18 ± 1039.71	393.85 ± 117.39
2 d	69.17 ± 12.86	74.05 ± 2.07	120.69 ± 5.97	2222.48 ± 575.86	121.64 ± 36.10	1267.44 ± 105.11	379.40 ± 15.06
3 d	89.69 ± 8.20	48.70 ± 14.16	69.05 ± 38.09	2917.96 ± 94.50	124.04 ± 79.02	175.89 ± 72.43	281.00 ± 39.21
7 d	50.59 ± 9.83	34.57 ± 15.12	57.76 ± 5.56	2855.88 ± 103.95	104.81 ± 36.22	185.15 ± 84.02	113.50 ± 13.57
10 d	40.66 ± 1.42	69.56 ± 2.56	52.04 ± 11.38	2312.21 ± 271.03	75.79 ± 32.27	107.79 ± 22.59	188.66 ± 54.17
14 d	45.46 ± 6.19	39.00 ± 7.30	55.87 ± 6.34	2082.92 ± 3.01	107.81 ± 47.28	138.94 ± 33.54	244.37 ± 86.09
17 d	34.21 ± 6.50	44.68 ± 4.56	52.13 ± 3.16	1514.49 ± 498.58	110.81 ± 34.44	118.92 ± 26.26	137.41 ± 9.64
21 d	23.49 ± 0.77	57.85 ± 1.74	59.52 ± 3.78	904.25 ± 295.32	108.66 ± 50.46	-	-

Notes: “ND” indicates that less than LOQ. “-” indicates no detected.

**Table 8:** Elimination parameters of deltamethrin residues in laying hens samples.

Sample	Elimination Equation	Elimination Rate (t^−1^)	Half-Life (Day)
Liver	y = 126.8 × 10^−2.258t^	2.258	0.3069
Heart	y = 240.7 × 10^−0.5597t^	0.5597	1.238
Kidney	y = 226.4 × 10^−0.7101t^	0.7101	0.9761
Lung	y = 3845 × 10^−3.328t^	3.328	0.2083
Fat	y = 3230 × 10^−0.04078t^	0.04078	17.00
Blood	y = 248.5 × 10^−0.08435t^	0.08435	8.217
Hen manure	y = 13,480 × 10^−1.557t^	1.557	0.4451
Eggs	y = 408.1 × 10^−0.07458t^	0.07458	9.294

## Data Availability

The data used to support the findings of this study can be made available by the corresponding author upon request.
